# Guarding the Portals of Entry

**Published:** 1918

**Authors:** Franklin W. Bock

**Affiliations:** Rochester, N. Y.


### GUARDING THE PORTALS OF ENTRY

**By Franklin W. Bock, M.D., Rochester, N. Y.**

No associated group of organs is of more practical importance to the growing child than the nose and mouth. They are frequently subject to all sorts of use and abuse, and when trouble arises we have little commendatory to say for them. It is remarkable how little thought until quite recently has been given to these organs which have such an important bearing upon the life and wellbeing of the child. Not only do the great quantities of fuel and nutriment enter the body by these portals, there being partially prepared for use, but here also enter and begin most of the infections and ills to which the young child is heir. It is not possible to discuss in detail all the phases of this subject which have a bearing upon the preservation of the child in health and his care during illness. I shall, therefore, confine myself to the broad discussion of the nose and mouth of the child from four points of view: their functional, pathological, and esthetic importance, and their relation to the special senses.

While the size and shape of the mouth and the integrity of the muscle groups which enter into its structure are of importance in the preparation of food for its entry into the stomach the mucous membrane with its associated glandular system is the most important part from the functioning and the pathological point of view. The secretions of these tissues are essential in the chemical changes which take place in early digestion, and under normal conditions they probably inhibit the growth of disease producing bacteria. A normal condition may be said to exist only when nothing but the normal secretions of the mucous membranes and glands are present in the mouth. This state of affairs unfortunately seldom obtains in the mouth of a human being after birth, for the products of the partial digestion and decomposition of food are usually present to greater or less extent, forming conditions which stimulate the growth of pathogenic germs. These, with the products of chemical change, sooner or later have a deleterious effect upon the integrity of mucous membranes and the underlying glands leading to almost any degree of functional or pathological change. The products of these varying degrees of pathologicol change enter the circulation through the stomach or lungs or directly through the submucous glandular system, producing far reaching and often obscure systematic effects.

I believe the only practical way of preventing these local and general ill effects is by keeping the mouth as free as possible from the products of chemical or bacterial activity. Cleanliness of the mouth is a fundamental principle in maintaining the young child in health, and this is best accomplished by the free use of pure water both as a drink and as a mouth wash. Pure water and plenty of it is quite as important to the young child as the quality of its food.

At about the first year a complicating element enters the child's mouth in the first teeth. These make cleanliness a more difficult problem, but it should be solved neverthless by tender and watchful care. Food should be washed from between the teeth after each meal and great care should be taken not to injure the delicate tissues around the teeth. I do not believe it is necessary, nor do I believe it is wise, to use the popular so called antiseptic mouth washes in a child's mouth. Plain water, or at most a normal salt solution, is all that is necessary to preserve the mouth of the child in a healthful condition.

While the membranes of the mouth have a wide range of accommodation probably without deleterious results, I do not believe it is wise to subject a child's mouth to too great extremes of temperature or to food which is too strongly seasoned. Irritation of this kind keeps up a low grade of congestion which finally has a bad effect upon the nutrition of the membranes. The introduction, however, of such fruits as lemons, oranges, and grapes into the dietary of the young child will go far toward keeping the mouth and teeth clean. If during the first two years of life decay appears in any of the teeth they should, of course, be attended to immediately, for each point of decay forms a focus of local or general infection. If cleanliness is a first principle in keeping the mouth of the child in a healthful condition, it is a paramount principle in diseased conditions, local or general, and it should be secured in the gentlest possible manner.

The function of the mucous membrane and the underlying erectile tissues of the nose and throat is to warm, moisten, and filter the air before it enters the lungs. If the air we breathe was clean I believe the nose would accomodate itself to great extremes of moisture and temperature without injuring its functioning ability to any great extent or producing pathological conditions, but unfortunately air is usually laden with irritating and infected dirt and dust, and the tissues of the nose are unable to handle this continuously without sooner or later becoming the victim of a low grade of inflammation with all the attending functional and pathological changes, which in common parlance we call catarrh. This condition of the mucous membrane of the nose and throat is to my mind the inevitable result of requiring it to filter more irritating and infected dirt than it is able to. How best to relieve this continuous overstrain is a problem of great importance, especially to the young child.

Many foolish things are done with young babies which better sense should interdict. A baby is not many months old before he is set down on the floor, where he cannot fall. And then we wonder why the baby's nose sems irritated and why he takes cold. The surprising thing is that babies stand the strain as well as they do. Young babies should not be allowed to creep around on the floor, not so much because of the draughts, but because of the dust that we and they stir up and then they inhale. Even under the best conditions babies have to inhale more dust and dirt than is good for their delicate membranes, and it is a good practice to wash it out of the baby's nose once in a while with a little normal salt solution.

This practice has been objected to by some on the ground that it will injure the delicate ciliated membranes. This may be so, but it would seem to be a matter of choosing the lesser of two evils, for certainly the washing cannot be nearly so harmful as the dirt mixed with mucus, which would otherwise pile up in the corners to form a splendid culture medium for the rapid growth of virulent germs. A few drops of normal salt solution from a medicine dropper will soon be tolerated by most babies, and it keeps the nose in better condition and helps to prevent infection.

Young children should be taught early to blow the nose properly, with both nostrils open, and not violently with one or both nostrils closed tight. The increase of intranasal pressure caused by improper methods of blowing the nose may force infected matter into the Eustachian tubes with disastrous results. Again, as in the mouth, the so called antiseptic washes should not be used in a small child's nose. Warm normal salt solution is the remedy par excellence both in preventive and curative practice to use in the baby's nose. Where the turgescence of the tissues is so greaat as to entirely block the nse, an astringent, such as adrenalin, should be used first, so the washing may be done more effectively. Astringents of any kind, however, should be used with the greatest care.

The prophylactic hygiene of the nose is not complete without mention of the ill effects with rapid extreme variations in the temperature of the body, general and local, have upon the child's nose and how best to guard against them. Chilling of the body, especially local chilling, almost instantly causes a sympathetic compensatory hypertrophy or engorgement of the erectile itssues of the nose and throat, producing what might be called a reflex cold, with difficult nasal respiration, excessive secretion, irritation, and sneezing. In children in normal health and vitality the moment the equilibrium of body temperature is reestablished the tissues of the nose and throat return to normal and no lasting ill effects are produced. If, however, the equilibrium is not reestablished within a time which varies in individuals, conditions are produced which make the rapid development of the germs always present in the nose and mouth more possible and probable, and added to the reflex cold we have an actual infective condition with all its possible far reaching and dangerous results.

It is this very rapid response to changes of temperature that makes it advisable during acute infective fevers to keep the child in as equable a temperature as possible. It matters little whether it is cool or warm as long as it is reasonably constant. Obviously the way to prevent chilling of the body is to pay more attention to the manner of clothing young children, especially the feet, legs, and arms. It is all very well to talk of the "hardening" process to parents, but if a child loses his hearing during the "hardening" there is little satisfaction in the result. I believe the actual fact is that a child cannot be "hardened." If he manages to live through it without serious mishap it is usually in spite of it and not because of it.

The care of the baby's nose and mouth in pathological conditions has been hinted at in the methods outlined for preserving the functional integrity of these organs. Cleanliness I believe, is the paramount consideration, and except in rare cases nothing stronger than normal salt solution should be used to secure it.

The effect of infectious diseases upon all the mucous membranes of the body, and especially of the nose and throat, is well known; very often they involve the middle ear, going on to abscess formation almost surely resulting in more or less reduction of hearing, and even sometimes ending in brain abscess and death. Even if the inflammation does not result in abscess or an immediate permanent reduction in hearing, conditions originate during infectious fevers and common colds which sooner or later surely bring on progressive middle ear deafness.

While I am not prepared to say that every case of middle ear involvement occurring during an infectious fever is the result of improper care, for it will sometimes come in spite of the best of care, I do believe that we would have less trouble of this kind and certainly fewer disastrous results if more attention were given to the cleanliness of the nose during acute infections. During the infectious fevers the ears should be examined daily or oftener for the first indications of trouble, and when it comes, if in doubt, always incise. Do not stop at treating the ear; keep the nose clean if it has not been attended to before.

We should not forget that both in prophylaxis and in cure we probably have a very powerful adjunct to our armamentarium in the vaccines. Only experience will demonstrate their correct value. However, it is my opinion that they should be used as an adjunct and never as the only treatment.

In discussing the pathology of the nose and mouth of the child one is drawn irresistibly to the problem and the enlarged and diseased faucial, pharyngeal, and lingual tonsils. While there seems to be doubt in the minds of some as to their function, it is inconceivable that the Almighty should have put them there just to make trouble for the child. In their normal state they are probably very efficient sentinels guarding these two great portals of entry against infection. Unfortunately, under the average present day personal and public hygiene they do not long remain normal. Uder the strain of violent and often overwhelming chemical and bacterial irritation they first enlarge to meet the extra work and finally and inevitably break down under the strain and themselves become diseased. In this state they affect the child deleteriously in two ways; by mechanically obstructing the respiration and circulation, producing more or less serious congestive conditions, and further by being culture beds for all grades of acute or chronic local or systemic infections.

Young children should be watched carefully for the first symptoms of these conditions and when they occur, if not improved by treatment the tonsils should be removed by surgical means. Age is not a contraindication for the operation when it is needed. It should always be treated as a major operation and done as carefully and with as little loss of blood as possible.

Earache or loss of hearing during colds is a positive indication for radical measures. A persistent postoperative system of mouth and nose hygiene will most certainly enhance the good results of operation or treatment.

Inherited and acquired syphilis may produce terrible havoc in the child's nose and mouth, and when discovered it should be treated quickly and vigorously.

The functional integrity of the special senses of taste and smell very decidedly upon a healthful condition of the mouth and nose, but one of the great economic social problems of the day is how best to safeguard the special sense of hearing. This, I believe, can only be done by keeping the mouth and nose in as healthful a condition as possible, by persistent and careful attention during acute local or general infections, and I include in this common colds.

The esthetic value of a healthy mouth and nose presents a subject of great interest. Every parent wants a good looking child, but how few parents and how few doctors realize that the regular or irregular development of the face in later childhood depends in large measure upon the care which the nose and mouth have received during the first few years of life, and especially during the first year of dentition?

A slight preventable or easily remediable irregularity of the first teeth may become an exaggerated and unfortunate deformity of the face or jaw, often ending in a high palatal arch with deformity of the nasal septum, producing a nasal deformity or obstruction to easy nasal respiration or both. While tonsils and adenoids are not entirely blameless, I believe faulty dental conditions are most often the chief causative factor. However, there is a vicious circle here which can most certainly be broken into by proper mouth and nose hygiene during these early years, or by the institution of simple orthodontic procedures at the proper time.

The maintenance of the child in health and the prevention of the spread of infection calls imperatively for a comprehensive system of mouth and nose hygiene. What this system is eventually to be is hard to tell, but I believe it will contain some at least of the following means of public and personal attack upon the problem: Young children should not be put upon the floor to play or creep. Next to the floor thousands of our children have no place to play but the street; hence have our streets kept in better condition. Young children should not be allowed to fondle domestic animals and pets. Cats and dogs might well be eliminated from our family life. Young children should be trained to drink more pure water. They should be trained to keep theid hands clean and to keep their fingers out of their nose and mouth. They should be trained to keep things out of their mouth. They should be trained always to wash their hands before eating. They should be taught early to tolerate and use a simple system of mouth and nose toilet.

These things are more easily said than done, but they should be done if the problem of keeping the young child well is to be solved, and that is the work of the medical man.—New York Medical Journal.

